# The Safety of Human Chorionic Gonadotropin Monotherapy Among Men With Previous Exogenous Testosterone Use

**DOI:** 10.7759/cureus.25826

**Published:** 2022-06-10

**Authors:** Quinn Rainer, Raghav Pai, Isaac Zucker, Ranjith Ramasamy, Thomas A Masterson

**Affiliations:** 1 Urology, University of Miami, Miami, USA; 2 Medicine, Florida International University, Miami, USA

**Keywords:** testosterone hormone, testosterone deficiency, hypogonadism, testosterone therapy, human chorionic gonadotropin

## Abstract

Background and objective

Human chorionic gonadotropin (hCG) is homologous to luteinizing hormone (LH) and stimulates endogenous testosterone (T) production. Current American Urological Association (AUA) guidelines recommend hCG for T-deficient men who wish to preserve their fertility. However, there is no data available regarding the long-term efficacy and safety of hCG monotherapy in men with a history of exogenous T use. We hypothesized that transitioning to hCG would be a safe and effective option in this population.

Methodology

We performed a retrospective analysis involving 28 men with previous exogenous T use who were switched to hCG monotherapy and underwent follow-up lab work at least one month later. We evaluated changes in hormones [T, LH, follicle-stimulating hormone (FSH), and estradiol], hematocrit (HCT), glycated hemoglobin (HbA1c), and prostate-specific antigen (PSA).

Results

Among the entire cohort, we found no significant change in mean hormone levels (including T), HbA1c, or PSA. There was a significant (p<0.05) decrease in HCT (45.27 ±4.06 to 44.16 ±3.48%, n=15). No thromboembolic events were reported. Additionally, among men who had their baseline labs completed outside their previous T therapy therapeutic time window prior to starting hCG monotherapy, there was a statistically significant increase in mean T levels (307.36 ±148.74 to 422.11 ±268.15 ng/dL, n=30 and 31, pre- and post-hCG, respectively) and a statistically significant decrease in mean PSA levels (0.91 ±0.35 to 0.69 ±0.23 ng/mL, n=5).

Conclusions

These results suggest that hCG is a safe and effective alternative to traditional T therapy for men with a history of exogenous T use and may lead to an advantageous decrease in HCT. hCG may serve as an alternative form of T therapy with a lower risk for secondary erythrocytosis, and further research is warranted to gain deeper insights into the topic.

## Introduction

Testosterone (T) therapy and anabolic steroid use are on the rise, with an estimated prevalence of four million users in the United States [1-3]. While effective at increasing serum T and relieving hypogonadal symptoms, exogenous T suppresses the hypothalamic-pituitary-gonadal axis, resulting in decreased luteinizing hormone (LH) and follicle-stimulating hormone (FSH) production from the anterior pituitary [4]. This, therefore, reduces Leydig and Sertoli cell stimulation, resulting in low intratesticular T and sperm production. Taken together, men experience infertility and testicular atrophy [5-7]. The most common dose-limiting side effect of exogenous T is increased hematocrit (HCT) levels, which may require dose adjustment, therapy cessation, or therapeutic phlebotomy [8,9]. Recent data suggest that men who experience T-induced polycythemia (more specifically, erythrocytosis) have an increased risk of venous thromboembolic events (VTE) and major adverse cardiovascular events (MACE) [10-12].

Human chorionic gonadotropin (hCG) is homologous to LH and stimulates endogenous T production from the testes. This serves as an alternative to exogenous T for men with secondary hypogonadism and increases serum T levels. hCG is produced by the human placentas and is derived from the urine of pregnant women or produced in vitro using recombinant DNA technology [13]. The American Urological Association (AUA) guidelines recommend hCG for T-deficient men with fertility concerns, as it prevents azoospermia seen in up to 60% of men on exogenous T [14]. However, there is very little data regarding the ability of hCG to maintain T in men with a history of exogenous T use, and its effect on reproductive hormones, prostate-specific antigen (PSA), and HCT have not been well studied. We hypothesized that hCG is an effective and safe treatment option in males with a history of exogenous T use. Our objective was to evaluate the changes in hormones, HCT, glycated hemoglobin (HbA1c), and PSA, as well as the side effects in men using hCG monotherapy with a history of exogenous T use.

## Materials and methods

Ethical approval

This study was conducted after obtaining the institutional review board (IRB) approval for retrospective chart review (IRB#: 20170849).

Inclusion and exclusion criteria

We included males with previous exogenous T use (including intramuscular injections, nasal gels, transdermal gels, and subcutaneous pellets) who visited our urology clinic between August 2015 and October 2021. They had stopped exogenous T use and had been subsequently placed on hCG monotherapy for at least one month with follow-up labs. Patients were excluded if they were on other forms of T therapy (such as clomiphene, anastrozole, or T) concurrently with hCG or did not have a follow-up testosterone laboratory result after at least one month.

Study design

We retrospectively analyzed the charts of 28 men with previous exogenous T use who visited our urology clinic and were subsequently on hCG monotherapy for at least one month with follow-up labs.

Data collection

We evaluated changes in hormones (T, LH, FSH, and estradiol), HCT, HbA1c, and PSA before and after initiating hCG monotherapy. When available, two pre- and post-hCG T serum levels were included for each patient. Additionally, it was noted if their pre-hCG/baseline labs were collected while the patient was using exogenous T. Men who were within the expected therapeutic time window of their respective T therapy for baseline labs when switching from T to hCG were categorized as baseline “On T” for the purpose of data analysis but were not excluded. The timing of these therapeutic ranges was based on Shoskes et al.’s paper [15]. For example, nasal gels were considered subtherapeutic after 500 minutes (five half-lives), intramuscular T cypionate was considered subtherapeutic after 14 days, and subdermal pellets were considered subtherapeutic after 12.5 months (five half-lives). We also conducted a chart review to determine VTE and MACE incidence, including stroke, deep vein thrombosis, and myocardial infarction.

Statistical analysis

All data analysis was performed in Excel (Version 2108, Build 14326.20404, Microsoft Corporation, Redmond, WA) and Stata (Version 17.0, StataCorp, College Station, TX). Results are presented as means and standard deviations. The Student's t-test analysis was used to compare pre- and post-treatment values; statistical significance was set at p<0.05.

## Results

Twenty-eight men who had a history of exogenous T use were included in the study. The mean age of the patients was 43.79 ±9.74 years (range: 24-66 years) with a mean BMI of 29.13 ±3.95 kg/m^2^ (range: 23.96-39.46 kg/m^2^). Demographic information based on the status of patients being on or off T for their pre-hCG monotherapy labs can be seen in Table 1. Most patients were started on hCG monotherapy due to fertility concerns (n=13). The second most common reason entailed men who presented with low T and a history of exogenous T use (n=9). Other, less common reasons included side effects of exogenous T therapy (n=4), a desire to be off exogenous T (n=1), and persistent symptoms despite T therapy (n=1). The mean duration between exogenous T cessation and the initiation of hCG monotherapy was 255.04 ±313.20 days (range: 0-1095 days, n=26). Eight of these patients began hCG monotherapy within a week of stopping exogenous T use, eight more patients began within a year of T cessation, and 10 patients began a year or more after T cessation. Eight patients were found to have completed “baseline” T levels within the therapeutic time window of their respective T therapy.

**Table 1:** Demographic data and hCG treatment details and number of patients based on the route of administration of previous exogenous T Grouped according to patients who were taking (“On T”) or not taking (“Off T”) exogenous T during their pre-hCG/baseline labs. The “On T” group had their baseline labs completed within the therapeutic window of their respective previous exogenous T therapy hCG: human chorionic gonadotropin. T: testosterone. BMI: body mass index. IM: intramuscular. TD: transdermal. SD: standard deviation

T therapy status at the time of baseline labs	Age, years, mean ±SD	BMI, kg/m^2^, mean ±SD	T cessation to hCG initiation, days, mean ±SD	Duration of hCG treatment, days, mean ±SD	IM injection, n (%)	Nasal gel, n (%)	TD gel, n (%)	Pellets, n (%)
All patients (n=28)	43.79 ±9.74	29.13 ±3.95	255.04 ±313.20 (n=26)	340.61 ±243.03	17 (61%)	6 (21%)	3 (11%)	2 (7%)
On T (n=8)	43.13 ±8.63	30.15 ±4.03	-	372.50 ±295.99	4 (50%)	2 (25%)	1 (12.5%)	1 (12.5%)
Off T (n=20)	44.05 ±10.63	28.66 ±3.83	268.39 ±316.29 (n=18)	327.85 ±225.95	13 (65%)	4 (20%)	2 (10%)	1 (5%)

The mean follow-up period after starting hCG therapy was 340.61 ±243.03 days (range: 50-936 days). The majority of patients were followed up for more than six months (≤6 months=9 patients, >6 months=19 patients). Twenty-one patients (75%) injected hCG once weekly, six patients (21%) injected twice weekly, and one patient (4%) injected three times weekly. Complete hCG dosage schedules of the patients included can be seen in Table 2. Of note, 21 of the 28 (91%) patients reported erectile dysfunction, low libido, and low energy at the time of presentation to the clinic. Of the patients reporting these symptoms, 15 (71%) reported improvement of their respective symptoms as compared to off T therapy.

**Table 2:** hCG dosing regimen of patients Analyzed as an entire cohort (“All Patients”) and by their status of baseline labs having been collected while a patient is on or off T hCG: human chorionic gonadotropin. T: testosterone

hCG dosing frequency	hCG dosing strength (IU)	All patients (n=28)	Baseline on T (n=8)	Baseline off T (n=20)
Weekly (n=21)	1000	2	-	2
	1500	11	1	10
	2000	8	3	5
Twice weekly (n=6)	1500	1	-	1
	2000	5	3	2
Thrice weekly (n=1)	2000	1	1	-

**Figure 1:**
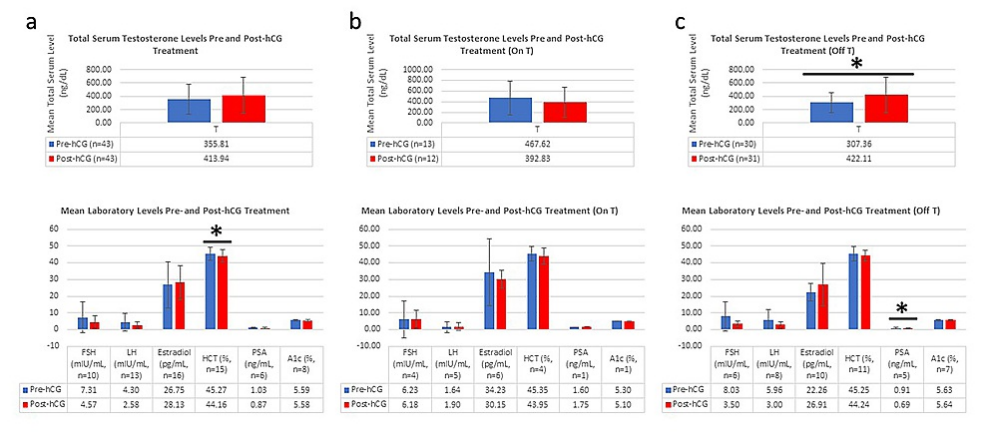
Bar graphs depicting mean laboratory results at the last available appointment pre-(blue) and post-(red) hCG monotherapy *Statistically significant (p<0.05) difference between mean pre- and post-hCG lab levels Error bars represent standard deviation. For the entire cohort (a, n=28), there was a statistically significant decrease in mean HCT from 45.27 ±4.06 to 44.16 ±3.48% (n=15). There was no statistically significant change in any lab results for patients "On T" at the time of their pre-hCG/baseline labs (b, n=8). For patients who were "Off T" at the time of their pre-hCG/baseline labs (c, n=20), there was a statistically significant increase in mean T from 307.36 ±148.74 to 422.11 ±268.15 ng/dL (n=30 and 31, respectively) and a statistically significant decrease in mean PSA from 0.91 ±0.35 to 0.69 ±0.23 ng/mL (n=5) hCG: human chorionic gonadotropin. LH: luteinizing hormone. FSH: follicle-stimulating hormone. HCT: hematocrit. HbA1c: glycated hemoglobin. PSA: prostate-specific antigen. T: testosterone

Pre- and post-hCG treatment laboratory results are displayed in Figure 1. The corresponding labs completed by each patient differed, but every patient included had at least one recorded T level pre- and post-hCG monotherapy. When the entire cohort was analyzed together (Figure 1A, n=28), mean serum T displayed a nonsignificant increase at the last follow-up with hCG monotherapy, and no change was seen in FSH, LH, PSA, estradiol, or HbA1c. There was a statistically significant decrease in HCT (45.27 ±4.06 to 44.16 ±3.48%, n=15). No VTEs or MACEs were observed. When the subgroup of patients who were on T at the time of their pre-hCG baseline labs (Figure 1B, n=8) was analyzed, there was no statistically significant change in any lab result. When the subgroup of patients who were off T at the time of their pre-hCG baseline labs (Figure 1C, n=20) was analyzed, there was a statistically significant increase in mean T from 307.36 ±148.74 to 422.11 ±268.15 ng/dL (n=30 and 31, pre- and post-hCG, respectively) and a statistically significant decrease in mean PSA from 0.91 ±0.35 to 0.69 ±0.23 ng/mL (n=5).

## Discussion

Exogenous T is not recommended for men interested in future fertility due to a high risk of developing azoospermia, and the AUA guidelines recommend hCG as an alternative. Unfortunately, there is very little safety data on men switching from T therapy to hCG. We set out to evaluate changes in serum hormone levels and side effects in men with a history of T therapy who switched to hCG monotherapy by performing a retrospective analysis. We found that T, reproductive hormones (LH, FSH, and estradiol), HbA1c, and PSA levels were maintained. Significantly, we also noticed a statistically significant decrease in HCT.

Our study consisted of a variety of hCG dosing regimens. This is largely due to shared decision-making between the physician and patient when considering their exogenous T use history, goals (treating infertility, treatment of symptoms, etc.), and comfort injecting. It should be noted that exogenous hCG has a half-life of 24-36 hours and men wishing to preserve their fertility typically require more frequent and stronger doses [16,17].

Using hCG as a strategy for increasing serum T is well known. A 1992 study by Vicari et al. using 1500 IU hCG three times weekly for 24 months in 17 men, with both large and small testes, demonstrated a statistically significant increase in plasma T levels [18]. Another study by Kim et al. in 2011 achieved similar results by administering 1500 to 2000 IU three times weekly for eight weeks to 20 men with hypogonadotropic hypogonadism [19]. They found a significant increase in mean serum T levels from 0.90 ±1.35 to 5.58 ±1.75 ng/mL after 24 weeks. While both studies indicate that hCG can successfully increase T levels in T-naive men, hCG's ability to maintain T levels in men who switch from exogenous T and its effect on reproductive hormones, PSA, and HCT have not been well studied.

hCG is effective in recovering spermatogenesis in azoospermic and oligospermic men on T therapy. A case series by Wenker et al. examined men with severe oligospermia who received combination hCG (with clomiphene citrate, tamoxifen, anastrozole, and/or recombinant FSH) therapy and found the return of spermatogenesis or improved counts in 47 of 49 men (95.9%) [20]. Despite these promising studies, there is currently scarce data examining the long-term safety of hCG treatment, especially regarding laboratory changes [21]. The data presented here suggest that hCG is a safe and effective long-term T therapy option. hCG may serve as a potential method of T therapy in patients who do not qualify for exogenous T, even those with former T use.

The decrease in HCT observed in our small study is of particular interest as it may be advantageous for men on T therapy with problematic secondary erythrocytosis, although this was not directly studied. T therapy has historically been associated with MACE [22,23]. Ory et al. recently confirmed through a multi-institutional database study that secondary erythrocytosis while on T therapy is an independent risk factor for MACE and VTE during the first year of therapy [12]. The pathophysiology of T therapy causing erythrocytosis is suspected to be multifactorial, involving an increased erythropoietin setpoint, decreased hepcidin, and increased estradiol [24]. The different physiological effects of hCG versus exogenous T, resulting in unique changes in HCT, should be explored in future studies.

For patients who were outside the therapeutic window of their previous T therapy prior to having their pre-hCG/baseline labs collected, there was a statistically significant increase in mean T levels and a decrease in mean PSA levels. PSA is often tracked in patients receiving T therapy given the historical belief that T promotes prostate cancer growth; however, recent evidence and meta-analyses suggest that T therapy does not appear to significantly increase PSA levels or cause prostate cancer progression [25,26]. The decrease in PSA demonstrated here further supports hCG’s safety and the increase in T validates its effectiveness.

Data regarding the safety of hCG is scarce, but a 2015 prospective study by La Vignera et al. compared the safety of hCG to exogenous T therapies (intramuscular injections and transdermal gel) for the treatment of late-onset hypogonadism in 40 men, randomized into one of four groups, after six months of each respective therapy [27]. They found an increase in HCT and PSA among the hCG group, but each was significantly less than that seen in men treated with exogenous T. La Vignera’s findings suggest that hCG may offer a safer form of T therapy, which is consistent with our results.

Limitations of our study include its retrospective design, the relatively small sample size of 28 (with the status of labs varying at follow-up), the usage of a mixture of T therapy formulations, the variable time interval from T cessation to starting hCG monotherapy, and the inability to determine medication adherence. Although it appears that hCG therapy may decrease HCT, we did not specifically test this in men with secondary erythrocytosis. This finding is interesting and requires further investigation. The strength of this study is that it is the first to quantify the safety of hCG in patients with previous T therapy.

## Conclusions

hCG dosing appears to maintain T levels in men transitioning off exogenous T. Men with baseline labs completed outside their previous T therapy therapeutic time window before initiating hCG monotherapy had a statistically significant increase in mean T levels and a decrease in PSA levels. Among the entire cohort, HCT levels showed a small but statistically significant decrease, and no VTEs or MACEs were recorded. Secondary erythrocytosis is a common adverse effect of T therapy and can lead to MACEs and VTEs. While our study was limited by its small sample size, it suggests that hCG may be associated with less risk of secondary erythrocytosis. For men requiring T therapy who are at risk of secondary erythrocytosis, the use of hCG should be investigated further.
